# Characterization of Omicron BA.4.6, XBB, and BQ.1.1 subvariants in hamsters

**DOI:** 10.1038/s42003-024-06015-w

**Published:** 2024-03-15

**Authors:** Peter J. Halfmann, Kiyoko Iwatsuki-Horimoto, Makoto Kuroda, Yuichiro Hirata, Seiya Yamayoshi, Shun Iida, Ryuta Uraki, Mutsumi Ito, Hiroshi Ueki, Yuri Furusawa, Yuko Sakai-Tagawa, Maki Kiso, Tammy Armbrust, Sam Spyra, Ken Maeda, Zhongde Wang, Masaki Imai, Tadaki Suzuki, Yoshihiro Kawaoka

**Affiliations:** 1 Influenza Research Institute, Department of Pathobiological Sciences, School of Veterinary Medicine, University of Wisconsin-Madison, Madison, WI 53711 USA; 2 Division of Virology, Institute of Medical Science, University of Tokyo, Tokyo, 108-8639 Japan; 3 Department of Pathology, National Institute of Infectious Diseases, Tokyo, 162-8640 Japan; 4 The Research Center for Global Viral Diseases, National Center for Global Health and Medicine Research Institute, Tokyo, 162-8655 Japan; 5 Department of Veterinary Science, National Institute of Infectious Diseases, Tokyo, 162-8640 Japan; 6 Department of Animal, Dairy, and Veterinary Sciences, College of Agriculture and Applied Sciences, Utah State University, Logan, UT 84322 USA; 7 The University of Tokyo, Pandemic Preparedness, Infection and Advanced Research Center (UTOPIA), Tokyo, 162-8655 Japan

**Keywords:** SARS-CoV-2, Pathogens

## Abstract

During the Omicron wave, previous variants such as BA.2, BA.4, and BA.5 were replaced by newer variants with additional mutations in the spike protein. These variants, BA.4.6, BQ.1.1, and XBB, have spread in different countries with different degrees of success. Here, we evaluated the replicative ability and pathogenicity of BA.4.6, BQ1.1, and XBB clinical isolates in male Syrian hamsters. Although we found no substantial differences in weight change among hamsters infected with these Omicron subvariants, the replicative ability of BQ.1.1 and XBB in lung tissue was higher than that of BA.4.6 and BA.5. Of note, BQ.1.1 was lethal in both male and female transgenic human ACE2 hamsters. In competition assays, XBB replicated better than BQ.1.1 in the nasal turbinate tissues of female hamsters previously infected with Omicron BA.2. These results suggest that newer Omicron subvariants in the XBB family are still evolving and should be closely monitored.

## Abstract (web summary)

Omicron variants BQ.1.1 and XBB increased pathogenicity in wild-type hamsters, with BQ.1.1 showing higher mortality in human ACE2 transgenic hamsters compared to earlier Omicron variants..

## Introduction

Since COVID-19 was declared a pandemic over three years ago by the World Health Organization, there have been over 756 million confirmed cases globally and more than 6.8 million associated deaths (https://covid19.who.int/). Despite the introduction of medical countermeasures against COVID-19^1^, SARS-CoV-2, the virus responsible for COVID-19, persists and continues to impose huge public health burdens and have economic consequences worldwide. Driven by the emergence and spread of novel variants such as B.1.1.7 (Alpha) and B.1.617.2 (Delta), several epidemic waves of SARS-CoV-2 infections have occurred during the pandemic.

The first Omicron variant, BA.1 (B.1.1.529), in the last SARS-CoV-2 wave emerged in late 2021 with more than 30 amino acid substitutions, deletions, or insertions in its spike protein, raising concerns of reduced susceptibility to neutralizing antibodies and vaccine efficacy^2^. Since the emergence of BA.1, a diverse family of Omicron subvariants has evolved, some members of which emerged simultaneously with newly acquired amino acid substitutions in the spike protein. The Omicron subvariant BA.4.6 acquired the R346T and N658S spike substitutions along with N487D in some isolates, which distinguish it from its predecessor, BA.4 (Supplementary Fig. 1a). The BQ.1.1 subvariant, which emerged from the parental variant BA.5, also acquired R346T along with two additional substitutions (K444T and N460K) in the receptor-binding domain (RBD) of the spike protein (Supplementary Fig. 1b). More concerning has been the emergence of XBB, which possesses a recombinant spike protein derived from two BA.2 subvariants, BA.2.75 and BJ.1. XBB has an additional 13 amino acid changes in its spike protein, including five substitutions and one deletion in the N-terminal domain (V83A, H146Q, Q183E, V213E, G252V, and the Y145 deletion) and eight amino acid substitutions in the RBD (G339H, R346T, L368I, V445P, G446S, N460K, F486S, and F490S) (Supplementary Fig. 1c).

The evolution of these subvariants with specific substitutions in the spike protein have resulted in the loss of effectiveness of therapeutic monoclonal antibodies. The BA.4.6 subvariant is resistant to COV2-2196 + COV2-2130^3^, whereas BQ.1.1 and XBB are resistant to LY-CoV1404 (Bebtelovimab)^4^, the last therapeutic monoclonal antibody approved to combat COVID-19. Although there is accumulating evidence of loss of efficacy of therapeutics against these emerging subvariants^3–7^, knowledge is scant regarding the pathogenic potential of these latest SARS-CoV-2 strains. Syrian hamsters are highly susceptible to SARS-CoV-2 and serve as a useful animal model for the evaluation of their pathogenicity and countermeasures against COVID-19^8,9^. Here, we characterized BA.4.6, BQ.1.1, and XBB in Syrian hamsters and compared them with an early variant, B.1.617.2 (Delta), and previous Omicron subvariants.

## Results

### Infection of Syrian hamsters with Omicron subvariants

**Fig. 1:**
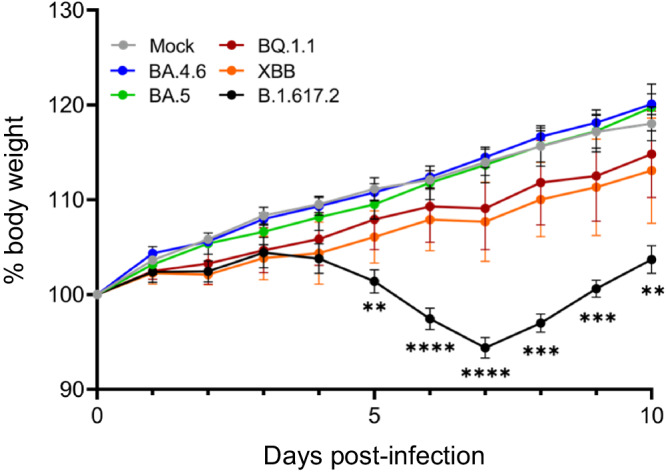
Body weight loss in wild-type Syrian hamsters infected with Omicron subvariants. Male hamsters (*n* = 5 per group) were infected intranasally with 10^5^ PFU (30 μL total volume) of BA.4 (UW12575 and UW12767), BQ.1.1, XBB, BA.5, or B.1.617.2, or with PBS (mock) and body weights were monitored daily for 10 days. Data are presented as the mean percentages of the starting weight (±s.e.m.). Data were analyzed by using a two-way analysis of variance (ANOVA) and Dunnett’s multiple comparisons test with the comparison to the mock-treated animals. *P* values: ** <0.01, *** <0.001, **** <0.0001.

The pathogenicity of Omicron subvariants [isolates of BA.4.6 (UW-12757), BQ.1.1 (TY41-796), and XBB (TY41-795)] was evaluated in wild-type male Syrian hamsters because male hamsters are more susceptibility to infection with SARS-CoV-2 variants than are female hamsters^10,11^. Here, we compared BA.4.6, BQ.1.1, and XBB with that of B.1.617.2 (Delta; UW5250) and an early Omicron subvariant, BA.5 (TY41-702). Male hamsters (*n* = 5 per group) were intranasally inoculated with 10^5^ plaque-forming units (PFU) of each variant and body weight was monitored for 10 days. Hamsters infected with the Omicron subvariants gained weight over the course of the study similarly to mock-infected animals, and there was no significant difference in body weight changes among the Omicron-infected hamster (Fig. 1). In contrast, infection with B.1.617.2 resulted in significant body weight loss compared to the other groups of hamsters starting at 5 days post-infection (dpi) with approximately 6% body weight loss at 7 days dpi (Fig. 1).

To assess the levels of infection in the respiratory tract of wild-type male Syrian hamsters, animals (*n* = 5 per virus/timepoint) were intranasally infected with 10^5^ PFU of B.1.617.2, BA.4.6, BA.5, BQ.1.1, or XBB. At 3 and 6 dpi, lung and nasal turbinate tissues were collected for virus titration on Vero E6-TMPRSS2-T2A-ACE2 cells by using the standard plaque assay.

**Fig. 2:**
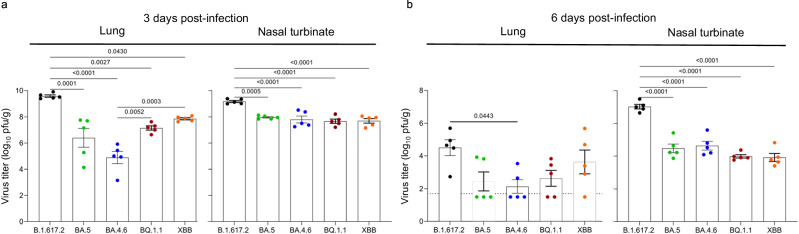
Growth kinetics of Omicron subvariants in wild-type Syrian hamsters. Male hamsters were infected with 10^5^ PFU (30 μL total volume) of BA.4 (UW12575 and UW12767), BQ.1.1, XBB, BA.5, or B.1.617.2. At 3 and 6 dpi, tissues were collected for virus titration (*n* = 5 per virus/timepoint). Virus titers in the lung (**a**) and nasal turbinate (**b**) tissues were determined by performing standard plaque assays with Vero E6-TMPRSS2-T2A-ACE2 cells. Vertical bars show the mean ± s.e.m. Points indicate data from individual hamsters. The lower limit of detection is indicated by the horizontal dashed line (**b**, lung). Data were analyzed by using a one-way ANOVA with Tukey’s multiple comparisons test. *P* values of <0.05 were considered statistically significant and are the only values shown.

As previously shown, the B.1.617.2 (Delta) variant grew more efficiently in the lung and nasal turbinate tissues at 3 dpi compared with the Omicron BA.5 variant (Fig. 2a)^12^. Similarly, the B.1.617.2 variant replicated more efficiently than all the other Omicron subvariants (BA.4.6, BQ.1.1, and XBB) evaluated here at 3 dpi in the lung [mean differences in viral titers 1.7–4.7 log_10_ (PFU/g)] and nasal turbinate tissues [mean differences in viral titers 1.0–1.5 log_10_ (PFU/g) (Fig. 2a). The BQ.1.1 and XBB variants replicated significantly better than the BA.4.6 variant in the lung tissues at 3 dpi (Fig. 2a). In the nasal turbinate tissues, all the Omicron subvariants replicated to similar titers at 3 dpi (Fig. 2a).

Virus titers were reduced at 6 dpi compared to the earlier timepoint in both tissues. Still, the B.1.617.2 variant replicated more efficiently in the lung and nasal turbinate tissues compared to the Omicron subvariants, with a statistically significant difference compared to one of the BA.4.6 isolates in the lung tissues (Fig. 2b). The XBB variant replicated more efficiently than the other Omicron subvariants [mean differences in viral titers 1.0–1.5 log_10_ (PFU/g)] in the lung tissues at 6 dpi, but the difference was not statistically significant (Fig. 2b).

### Histopathological findings in the lungs of Syrian hamsters infected with Omicron subvariants

**Fig. 3:**
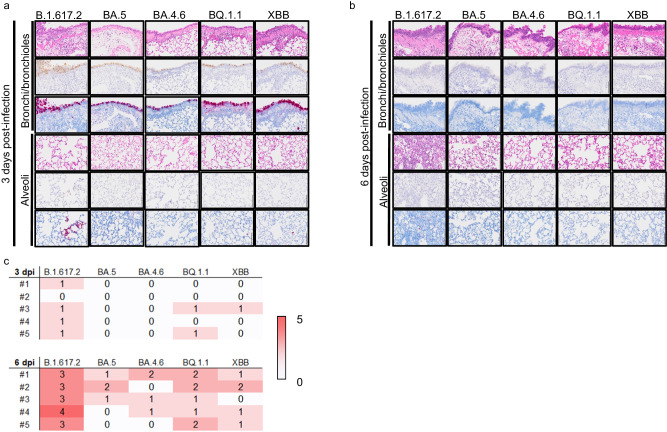
Histopathological findings in wild-type hamsters infected with Omicron subvariants. Male hamsters (*n* = 5 per virus/timepoint) were infected with 10^5^ PFU (30 μL total volume) of BA.4 (UW12575 and UW12767), BQ.1.1, XBB, BA.5, or B.1.617.2. Tissues were collected at 3 dpi (**a**) or 6 dpi (**b**) for histopathological examinations. Representative images of the bronchi/bronchioles and alveoli at high magnification are shown. Upper rows, hematoxylin and eosin staining. Middle rows, immunohistochemistry with a rabbit polyclonal antibody that detects SARS-CoV-2 nucleocapsid protein. Lower rows, in situ hybridization targeting the nucleocapsid gene of SARS-CoV-2. Scale bars, 100 µm. **c** Histopathological scores of inflammation in alveolar regions for all animals examined are shown. The scores were determined on the basis of the percentage of alveolar inflammation in a given area of a pulmonary section collected from each animal in each group using the following scoring system: 0, no inflammation; 1, affected area (≤1%); 2, affected area (>1%, ≤10%); 3, affected area (>10%, ≤50%); 4, affected area (>50%); an additional point was added when pulmonary edema and/or alveolar hemorrhage was observed. Therefore, histopathological scores of inflammations in alveolar regions for individual animal range from 0 to 5.

We performed a histopathological analysis of the lungs of wild-type male Syrian hamsters infected with BA.1.617.2, BA.5, BA.4.6, BQ.1.1, or XBB at 3 and 6 dpi (*n* = 5 per virus/timepoint). For the Omicron subvariants evaluated, we observed inflammation consisting of mononuclear cell and neutrophil infiltration in the peribronchial and peribronchiolar regions of the lungs at 6 dpi, but not at 3 dpi (Fig. 3a, b); no obvious alveolar inflammation was observed at 6 dpi in the lungs of animals infected with the Omicron subvariants (Fig. 3b). In contrast, the lungs obtained from animals infected with B.1.617.2 (Delta) showed considerable bronchial and peribronchial inflammation at 3 dpi and extensive pneumonia in the alveolar region at 6 dpi (Fig. 3a, b). Histopathological scores for inflammation in the alveolar region at 6 dpi were comparable between the BA.5 and BA.4.6 variants, whereas the scores were higher for hamsters infected with BQ.1.1 or XBB. However, the scores from all Omicron subvariant-infected hamsters were lower than those for B.1.617.2-infected hamsters at 6 dpi (Fig. 3c). In situ hybridization and immunohistochemistry demonstrated that viral RNA and antigen were readily detected in the bronchi/bronchial epithelium from hamsters infected with the Omicron subvariants at 3 dpi, but no longer detectable at 6 dpi (Fig. 3a, b). In the alveolar region of hamsters infected with the Omicron subvariants, few viral RNA- and antigen-positive cells were detected at 3 and 6 dpi (Fig. 3a, b). In contrast, viral RNA and antigen were detected diffusely in the bronchial/bronchopulmonary region and patchily in the alveolar region of hamsters infected with B.1.617.2 at 3 dpi. The number of viral RNA/antigen-positive cells decreased over time in both regions of animals infected with B.1.617.2 (Fig. 3a, b). Overall, there were clear differences in the histological findings in hamsters infected with the different Omicron subvariants, BA.4.6, BA.5, BQ.1.1, and XBB at 3 dpi or 6 dpi. These results suggest the Omicron subvariants tested here do not cause obvious pneumonia in the hamster model, although histology scoring based on alveolar inflammation is high in BQ.1.1- and XBB-infected hamsters. The number of viral RNA/antigen-positive cells in the alveolar region of animals infected with the Omicron subvariants was comparable to that in hamsters infected with an early Omicron subvariant (i.e., BA.5), but lower than in animals infected with B.1.617.2.

### Infection of human (h)ACE2 hamsters with XBB and BQ.1.1

**Fig. 4:**
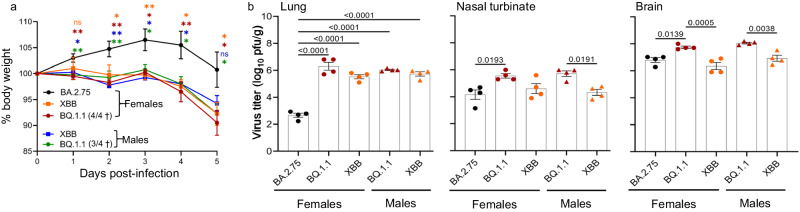
Growth kinetics of Omicron subvariants in hACE2 hamsters. Naïve transgenic hACE2 Syrian hamsters (*n* = 4 males and *n* = 4 females for BQ.1.1 and XBB; *n* = 4 females for BA.2.75) were infected with 10^5^ PFU (30 μL total volume) of virus. **a** Body weights of virus-infected hACE2 hamsters were monitored daily for 5 days. Data were analyzed by using a two-way analysis of variance (ANOVA) and Dunnett’s multiple comparisons test with the comparison to the BA.2.75-infected animals. *P* values: * <0.05, ** <0.01; ns - not significant. † - lethality in the indicated number of hamsters out of a total of four infected hamsters. **b** A separate group of infected animals was euthanized at 4 dpi for virus titration (*n* = 4 males and *n* = 4 females for BQ.1.1 and XBB; *n* = 4 females for BA.2.75). Virus titers in the lung, nasal turbinate, and brain tissues were determined by performing a standard plaque assay with Vero E6-TMPRSS2-T2A-ACE2 cells. Vertical bars show the mean ± s.e.m. Points indicate data from individual animals. The lower limit of detection is indicated by the horizontal dashed line. Data were analyzed by using a one-way ANOVA with Tukey’s multiple comparisons test. *P* values of <0.05 were considered statistically significant and are the only values shown.

We also investigated the infectivity of these Omicron subvariants in transgenic hamsters expressing hACE2. Given the greater susceptibility of these transgenic hamsters to SARS-CoV-2 infection relative to wild-type hamsters^12,13^, we used both male and female transgenic hamsters in this study. Animals (*n* = 4 males and *n* = 4 females per virus) were intranasally inoculated with 10^5^ PFU of each variant and body weight was monitored for 5 days. Animals infected with XBB or BQ.1.1 showed 10% body weight loss at 5 dpi (Fig. 4a). Strikingly, BQ.1.1 infection resulted in 100% mortality in females and 75% mortality in males at 5 dpi. In contrast, hACE2 hamsters infected with BA.2.75, an ancestor of XBB in the BA.2 sublineage, gained weight until 3 dpi and then returned to their original body weight at 5 dpi (Fig. 4a).

Virus titers at 4 dpi were determined in the lung, nasal turbinate, and brain tissues of hACE2 hamsters (*n* = 4 males and *n* = 4 females per virus). The virus titers in the lungs of hACE2 hamsters infected with BQ.1.1 or XBB were significantly higher than those in BA.2.75-infected hamsters [mean differences in viral titer = 3.6 and 2.9 log_10_ (PFU/g), respectively]. However, there was no statistically significant difference in virus titers in the lungs between the male and female hACE2 hamsters infected BQ.1.1 or XBB (Fig. 4b). In the nasal turbinate tissues, BQ.1.1 replicated to higher viral titers than BA.2.75 or XBB [mean differences in viral titers = 1.4 and 1.0 log10 (PFU/g), respectively], but was only significantly higher compared to BA.2.75 in female hamsters (Fig. 4b). In male hACE2 hamsters, BQ.1.1 replicated significantly better than XBB [mean differences in viral titers = 1.4 log_10_ (PFU/g)] in the nasal turbinates (Fig. 4b). The virus titers of BQ.1.1 in the brain tissues were significantly higher than those of BA.2.75 and XBB [mean differences in viral titer = 1.0 and 1.1 (PFU/g), respectively] in female hACE2 hamsters and significantly higher than those of XBB [mean differences in viral titers = 1.1 log_10_ (PFU/g)] in male hACE2 hamsters (Fig. 4b).

### The replicative fitness of Omicron subvariants in hamsters

Previously, we compared the replicative fitness in hamsters of Omicron variants by co-infecting naïve animals with a mixture of two variants and then determined their prevalence in nasal turbinate and lung tissues^12,14^. However, since many people have been infected with SARS-CoV-2 or vaccinated, it was important to compare replicative fitness in animals with pre-existing immunity. We, therefore, reinfected hACE2 female hamsters (*n* = 3 per BA.1- or BA.2-previously infected animals) that had been infected nine months previously with either BA.1 or BA.2 with a mixture of BQ.1.1 and XBB and examined virus in the nasal turbinate and lungs four days later.

**Fig. 5:**
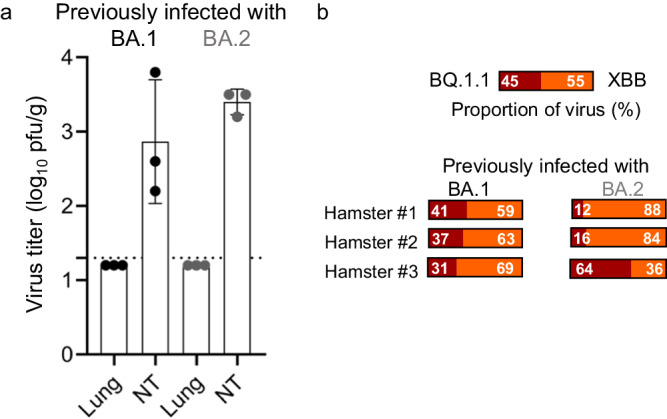
Relative viral fitness of Omicron subvariants in hamsters. **a** Virus titers of the mixture of BQ.1.1 and XBB at 3 dpi in the lung and nasal turbinate tissues of BA.1 or BA.2 previously infected female hACE2 hamsters (*n* = 4 per virus) were determined by performing a standard plaque assay with Vero E6-TMPRSS2-T2A-ACE2 cells. Vertical bars show the mean ± s.e.m. Points indicate data from individual animals. The lower limit of detection is indicated by the horizontal dashed line. **b** Using NGS, the relative proportions of BQ.1.1 and XBB from the nasal turbinate tissues of the infected animals were analyzed.

Infectious virus (BQ.1.1 or XBB) was not detected in the lungs, but we did detect virus in the nasal turbinate tissues (Fig. 5a). Therefore, NGS analysis was used to determine the proportion of virus in the nasal turbinate tissues. In animal previously infected with BA.1, the proportions of BQ.1.1 and XBB were similar in all three hamsters when compared to the inoculum (Fig. 5b). When BA.2 was used in the first infection, XBB became the dominant virus in two of three infected hamsters (Fig. 5b). These data suggest that in the background of preexisting immunity from a BA.2 infection, XBB may have great replicative fitness than BQ.1.1.

## Discussion

After the global dominance of the BA.5 Omicron subvariant, new variants with a cR346T substitution in the spike protein emerged, including BA.4.6, BQ.1.1, and XBB. Globally, there was an increase in the number of infections with BQ.1 and BQ.1.1 starting in late 2022. This increase was observed primarily in European countries like the United Kingdom and France, but also in the United States^15^. However, countries like India and Singapore observed an increase in cases caused by XBB infections^15^.

Since the emergence of Omicron BA.1, several new subvariants have arisen with an attenuated phenotype in animal models^16–19^. Here, we observed a similar attenuated phenotype among the Omicron subvariants BA.4.6, BQ.1.1, and XBB in Syrian male hamsters, although we noted some differences between BQ.1.1 and XBB. Wild-type male hamsters infected with BA.4.6, BQ.1.1, or XBB gained weight after infection comparable to hamsters infected with the early Omicron subvariant BA.5 or mock-infected animals (Fig. 1). In contrast, infection of transgenic hACE2 hamsters (both males and females) with BQ.1.1 or XBB resulted in a decline in body weight compared with those infected with the earlier Omicron subvariant BA.2.75 (Fig. 4a). In hACE2 hamsters, the higher replicative ability of BQ.1.1 compared with BA.2.75 and XBB, particularly in the brain, may explain the mortality associated with BQ.1.1 in these transgenic animals (Fig. 4b). In hACE2 hamsters, no significant differences were observed between male and female transgenic animals (Fig. 4a, b), although other groups have reported increased susceptibility to SARS-CoV-2 infections in wild-type male hamsters^10,11^. Ito et al. previously reported that the spike protein of BQ.1.1 has enhanced binding affinity to hACE2^20^, which may partially explain the better replication and lethality of BQ.1.1 in transgenic hACE2 hamsters.

Infection of wild-type hamsters with the BQ.1.1 or XBB isolate resulted in higher histology scores based on alveolar inflammation at 6 dpi compared with earlier Omicron subvariants such as BA.5 and BA.4.6 (Fig. 3c). However, the pathogenicity resulting from infection with any of the Omicron isolates in wild-type hamsters was not as pronounced as that induced by infection with the Delta (B.1.617.2) variant (Fig. 4a, b). The increases in histology scores may reflect the higher replicative ability of BQ.1.1 and XBB in the lung tissues of infected wild-type hamsters at 3 dpi compared to that of BA.4.6 and BA.5 (Fig. 2a).

Because of pre-existing immunity in human populations, we compared the replicative fitness of BQ.1.1 and XBB in female transgenic human ACE2 hamsters that were previously infected, 9-months prior, with either BA.1 or BA.2. In the context of pre-existing immunity, XBB with its greater number of amino acid substitutions in the spike protein was dominant over BQ.1.1 in the nasal turbinate tissues of hamsters previously infected with BA.2, but not BA.1 (Fig. 5). Because most human populations are now immune to SARS-CoV-2 through either infection or immunization, the Syrian hamster model with pre-existing immunity may provide a means to explore how prior immunity or lack thereof to different SARS-CoV-2 viruses may influence the replicative fitness of circulating variants.

In summary, while the BA.4.6 subvariant had similar attenuated characteristics to those of earlier Omicron isolates like BA.5, the BQ.1.1 and XBB subvariants replicate more efficiently in the lungs of hamsters with increased pathogenicity with a lethal phenotype of BQ.1.1 in transgenic human ACE2 hamsters. Given pre-existing immunity from vaccinations and previous infections, as well as underlying comorbidities, it is difficult to assess the disease phenotypes of BQ.1.1 and XBB in humans relative to our findings in hamsters. Cases studies in India indicate that the XBB variant causes mild disease^21^; however, no published data are available on the BQ.1.1 variant. As these subvariants acquire additional mutations, as XBB.1.5 has, the evolution of new sublineages must continue to be monitored for changes in vaccine susceptibility and overall virus pathogenicity.

## Materials and methods

### Cells

Vero E6/TMPRSS2 (JCRB 1819) cells were propagated in the presence of 1 mg/ml geneticin (G418; Invivogen) and 5 μg/ml plasmocin prophylactic (Invivogen) in Dulbecco’s modified Eagle’s medium (DMEM) containing 10% Fetal Calf Serum (FCS). Vero E6-TMPRSS2-T2A-ACE2 cells (provided by Dr. Barney Graham, NIAID Vaccine Research Center) were cultured in DMEM supplemented with 10% FCS, 10 mM HEPES pH 7.3, 100 U/mL penicillin–streptomycin, and 10 μg/mL puromycin. Vero E6/TMPRSS2 and Vero E6-TMPRSS2-T2A-ACE2 cells were maintained at 37 °C with 5% CO_2_. The cells were regularly tested for mycoplasma contamination by using PCR and confirmed to be mycoplasma-free.

### Viruses

The following viruses, BA.4.6^3^ [hCoV-19/USA/WI-UW-12757/2022 (UW-12757)], BQ.1.1 and XBB^4^ [hCoV-19/Japan/TY41-796/2022 (Accession ID; EPI_ISL_16355655) and hCoV-19/Japan/TY41-795 (Accession ID; EPI_ISL_16355653)] respectively, BA.2.75 [hCoV-19/Japan/TY41-716/2022 (Accession ID; EPI_ISL_14011362)], BA.5^12^ [hCoV-19/Japan/TY41-702/2022 (Accession ID; EPI_ISL_13512581)], and B.1.617.2 [hCoV-19/USA/WI-UW-5250/2021], were propagated in Vero E6/TMPRSS2 cells. All experiments with SARS-CoV-2 were performed in enhanced biosafety level 3 (BSL3) containment laboratories at the University of Tokyo and the National Institute of Infectious Diseases, Japan, which are approved for such use by the Ministry of Agriculture, Forestry, and Fisheries, Japan, or in BSL3 agriculture containment laboratories at the University of Wisconsin-Madison, which are approved for such use by the Centers for Disease Control and Prevention and by the US Department of Agriculture.

### Animal experiments and approvals

Animal studies were performed under protocols approved by the Animal Experiment Committee of the Institute of Medical Science, the University of Tokyo (approval number PA19-75) and the Institutional Animal Care and Use Committee at the University of Wisconsin, Madison (protocol number V006426). We have complied with all relevant ethical regulations for animal use. Virus infections were performed under isoflurane, and all efforts were made to minimize pain. Animal studies were not blinded, and animals were randomly assigned to infection groups. Group sizes were determined based on prior virus challenge studies, and no sample-size calculations were performed to determine the power of each study.

### Experimental infection of Syrian hamsters

Six-week-old male wild-type Syrian hamsters (Japan SLC Inc., Shizuoka, Japan) were used in this study. Baseline body weights were measured before infection. Under isoflurane anesthesia, hamsters (*n* = 5 per group) were infected by intranasal inoculation with the indicated virus isolates. Body weights were monitored daily for 10 days. For virological and pathological examinations, animals (*n* = 5 per virus/timepoint) were euthanized at the indicated timepoints. Lung and nasal turbinate tissues were collected for virus titrations, which were determined by using plaque assays on Vero E6-TMPRSS2-T2A-ACE2 cells.

Male and female 6–8-week-old K18-hACE2 homozygous transgenic hamsters^22^ (*n* = 4 males and *n* = 4 females per virus) were infected by intranasal inoculation with 10^5^ PFU of the indicated virus isolates. For acute challenge studies, body weights were monitored for five days. An additional group of animals was euthanized at 4 dpi and lung and nasal turbinate tissues were collected for virus titration. To examine replicative fitness with pre-existing immunity, females transgenic hACE2 hamsters (*n* = 3 per group) were intranasal inoculated with 10^5^ PFU of the indicated virus and then re-inoculated nine months later with a mixture of BQ.1.1 and XBB.

### Histopathology

Histopathological examination was performed as previously described^14,23,24^. In brief, excised animal lungs were fixed in 4% paraformaldehyde in phosphate buffered saline (PBS) and processed for paraffin embedding. The paraffin blocks were sliced into 3µm-thick sections and mounted on silane-coated glass slides, followed by hematoxylin and eosin (H&E) staining for histopathological examination. To detect SARS-CoV-2 RNA, in situ hybridization was performed using an RNA scope 2.5 HD Red Detection kit (Advanced Cell Diagnostics, Newark, California) with an antisense probe targeting the nucleocapsid gene of SARS-CoV-2 (Advanced Cell Diagnostics) an following the manufacturer’s instructions. Tissue sections were also processed for immunohistochemistry with a rabbit polyclonal antibody for SARS-CoV nucleocapsid protein (ProSpec; ANT-180, 1:500 dilution, Rehovot, Israel), which cross-reacts with SARS-CoV-2 nucleocapsid protein. Specific antigen-antibody reactions were visualized by means of 3,3’-diaminobenzidine tetrahydrochloride staining using the Dako Envision system (Dako Cytomation; K4001, 1:1 dilution, Glostrup, Denmark).

### Whole genome sequencing

Viral RNA was extracted by using a QIAamp Viral RNA Mini Kit (QIAGEN). The whole genome of SARS-CoV-2 was amplified by using a modified ARTIC network protocol in which some primers were replaced or added^25,26^. Briefly, viral cDNA was synthesized from the extracted RNA by using a LunarScript RT SuperMix Kit (New England BioLabs). The DNA was then amplified by performing a multiplexed PCR in two pools using the ARTIC-N5 primers and the Q5 Hot Start DNA polymerase (New England BioLabs)^27^. The DNA libraries for Illumina NGS were prepared from pooled amplicons by using a QIAseq FX DNA Library Kit (QIAGEN) and were then analyzed by using the iSeq 100 System (Illumina). To determine the sequences of BQ.1.1 and XBB, the reads were assembled by the CLC Genomics Workbench (version 22, Qiagen) with the Wuhan/Hu-1/2019 sequence (GenBank accession no. MN908947) as a reference. For the analysis of the ratio of viruses after co-infection, the proportions BQ.1.1 to XBB were calculated based on the differences in the spike protein at 6 positions between the pairs of viruses. Samples with more than 300 read-depths were analyzed.

### Statistical analysis and reproducibility

GraphPad Prism software was used to analyze the data. Statistical analysis included the Kruskal-Wallis test followed by Dunn’s test, and an ANOVA with post-hoc tests. Differences among groups were considered significant for *P* values < 0.05. Group sizes are noted in figure legends.

### Reporting summary

Further information on research design is available in the Nature Portfolio Reporting Summary linked to this article.

### Supplementary information

Supplementary Information

Description of Additional Supplementary Files

Supplementary Data 1

Reporting Summary

## Data Availability

All data supporting the findings in this study are available in the source data file and from the corresponding authors upon request. The numerical source data behind the graphs in Figs. 1, 2, 4, and 5 can be found in Supplementary Data 1.
