# Supplementary material for: Characterization of Omicron BA.4.6, XBB, and BQ.1.1 subvariants in hamsters
Source: Commun Biol. 2024 Mar 15;7:331. doi: 10.1038/s42003-024-06015-w (PMC10943235; doi:10.1038/s42003-024-06015-w)
Supplement: Supplementary file 1 — Supplementary Information [file 42003_2024_6015_MOESM1_ESM.pdf]

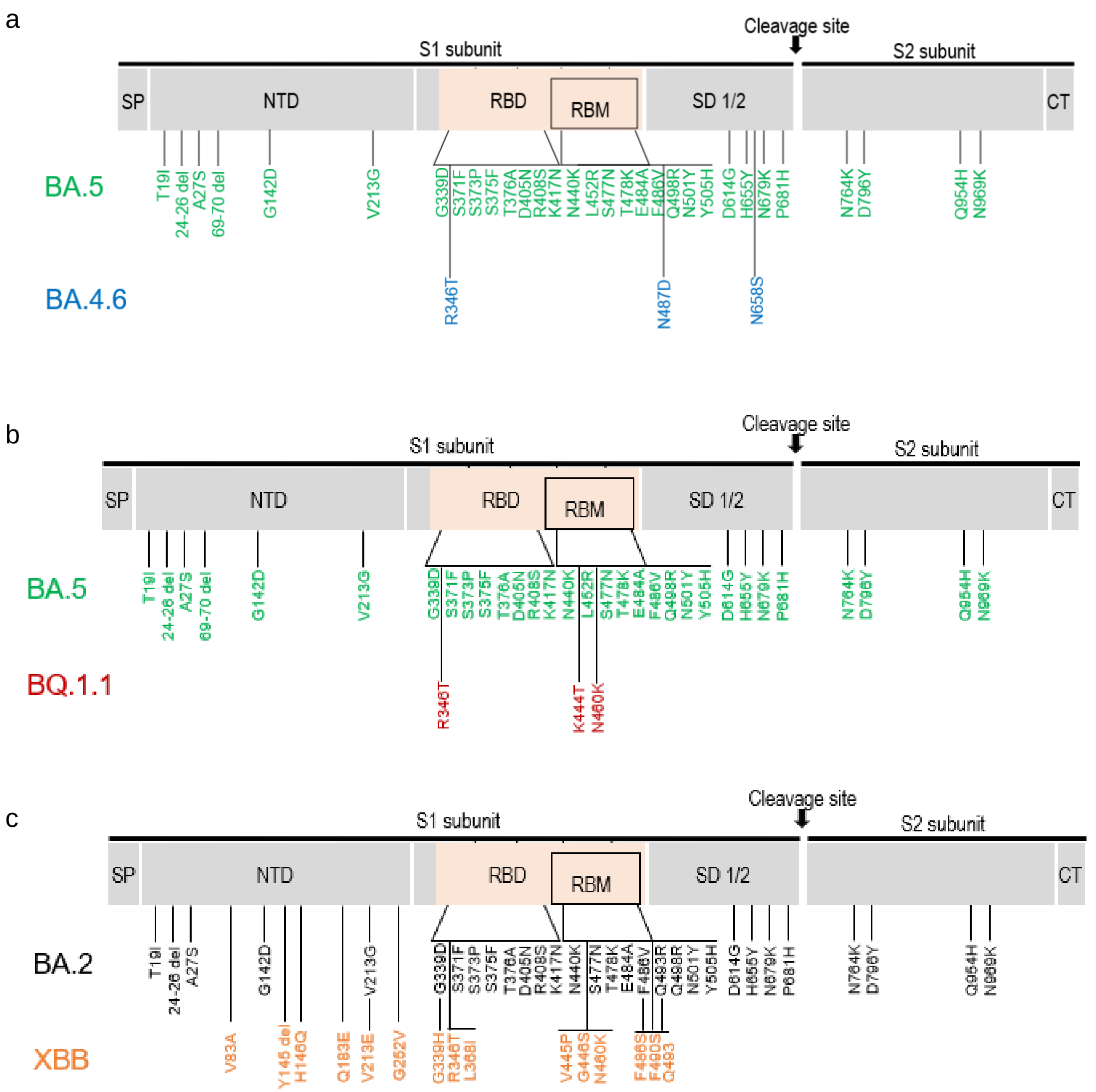

Supplementary Figure 1. Amino acid differences in the spike proteins between Omicron BA.5 and (**a**) BA.4.6 or (**b**) BQ.1.1 and between Omicron BA.2 and (**c**) XBB.
