# Supplementary material for: Characterization of Omicron BA.4.6, XBB, and BQ.1.1 subvariants in hamsters
Source: Commun Biol. 2024 Mar 15;7:331. doi: 10.1038/s42003-024-06015-w (PMC10943235; doi:10.1038/s42003-024-06015-w)
Supplement: Supplementary file 2 — Description of Additional Supplementary Files [file 42003_2024_6015_MOESM2_ESM.pdf]

Description of Additional Supplementary Files

File name: Supplementary Data 1
Description: The numerical source data behind the graphs in Figures 1, 2, 4, and 5.
