# Supplementary material for: Characterization of Omicron BA.4.6, XBB, and BQ.1.1 subvariants in hamsters
Source: Commun Biol. 2024 Mar 15;7:331. doi: 10.1038/s42003-024-06015-w (PMC10943235; doi:10.1038/s42003-024-06015-w)
Supplement: Supplementary file 4 — Reporting Summary [file 42003_2024_6015_MOESM4_ESM.pdf]

nature portfolio

Corresponding author(s): Halfmann

Last updated by author(s): 01-11-2024

Reporting Summary

Nature Portfolio wishes to improve the reproducibility of the work that we publish. This form provides structure for consistency and transparency in reporting. For further information on Nature Portfolio policies, see our Editorial Policies and the Editorial Policy Checklist.

Statistics

For all statistical analyses, confirm that the following items are present in the figure legend, table legend, main text, or Methods section.

n/a	Confirmed	
☐	☒	The exact sample size (*n*) for each experimental group/condition, given as a discrete number and unit of measurement
☒	☐	A statement on whether measurements were taken from distinct samples or whether the same sample was measured repeatedly
☐	☒	The statistical test(s) used AND whether they are one- or two-sided<br>*Only common tests should be described solely by name; describe more complex techniques in the Methods section.*
☒	☐	A description of all covariates tested
☒	☐	A description of any assumptions or corrections, such as tests of normality and adjustment for multiple comparisons
☐	☒	A full description of the statistical parameters including central tendency (e.g. means) or other basic estimates (e.g. regression coefficient) AND variation (e.g. standard deviation) or associated estimates of uncertainty (e.g. confidence intervals)
☐	☒	For null hypothesis testing, the test statistic (e.g. *F*, *t*, *r*) with confidence intervals, effect sizes, degrees of freedom and *P* value noted<br>*Give P values as exact values whenever suitable.*
☒	☐	For Bayesian analysis, information on the choice of priors and Markov chain Monte Carlo settings
☒	☐	For hierarchical and complex designs, identification of the appropriate level for tests and full reporting of outcomes
☒	☐	Estimates of effect sizes (e.g. Cohen's *d*, Pearson's *r*), indicating how they were calculated

Our web collection on statistics for biologists contains articles on many of the points above.

Software and code

Policy information about availability of computer code

Data collection	iSeq 100 System (Illumina), CLC Genomics Workbench (v22, Qiagen)
Data analysis	Prism 8.0 was used for the statistical analysis

For manuscripts utilizing custom algorithms or software that are central to the research but not yet described in published literature, software must be made available to editors and reviewers. We strongly encourage code deposition in a community repository (e.g. GitHub). See the Nature Portfolio guidelines for submitting code & software for further information.

Data

Policy information about availability of data

All manuscripts must include a data availability statement. This statement should provide the following information, where applicable:

- Accession codes, unique identifiers, or web links for publicly available datasets
- A description of any restrictions on data availability
- For clinical datasets or third party data, please ensure that the statement adheres to our policy

All data supporting the findings in this study are provided as a Source Data file and are available from the corresponding authors upon request.

Research involving human participants, their data, or biological material

Policy information about studies with human participants or human data. See also policy information about sex, gender (identity/presentation), and sexual orientation and race, ethnicity and racism.

Reporting on sex and gender	N/A
Reporting on race, ethnicity, or other socially relevant groupings	N/A
Population characteristics	N/A
Recruitment	N/A
Ethics oversight	N/A

Note that full information on the approval of the study protocol must also be provided in the manuscript.

Field-specific reporting

Please select the one below that is the best fit for your research. If you are not sure, read the appropriate sections before making your selection.

☒ Life sciences ☐ Behavioural & social sciences ☐ Ecological, evolutionary & environmental sciences

For a reference copy of the document with all sections, see nature.com/documents/nr-reporting-summary-flat.pdf

Life sciences study design

All studies must disclose on these points even when the disclosure is negative.

Sample size	No sample-size calculations were formed. No statistical method was used to determine the sample sized. Hamster studies used 3-4 animals per group. All sample sizes were chosen based on prior experience PMID:34140350 and 32571934.
Data exclusions	No data was excluded.
Replication	Because of USDA covered hamsters, each experiment was not carried out in duplicate.
Randomization	No method of randomization was used to determine how the animals were allocated to the experimental groups and processed in this study. However, covariates including sex and age were identical in groups.
Blinding	No blinding was carried out due to the limited number of staff available to conduct these studies. However, different researchers participated in different parts of each study (animal infections, weight collection, tissue collection, titrations, foci counting, and analysis

Reporting for specific materials, systems and methods

We require information from authors about some types of materials, experimental systems and methods used in many studies. Here, indicate whether each material, system or method listed is relevant to your study. If you are not sure if a list item applies to your research, read the appropriate section before selecting a response.

Materials & experimental systems

n/a	Involved in the study	
☐	☒	Antibodies
☐	☒	Eukaryotic cell lines
☒	☐	Palaeontology and archaeology
☐	☒	Animals and other organisms
☒	☐	Clinical data
☒	☐	Dual use research of concern
☒	☐	Plants

Methods

n/a	Involved in the study	
☒	☐	ChIP-seq
☒	☐	Flow cytometry
☒	☐	MRI-based neuroimaging

Antibodies

Antibodies used	Rabbit polyclonal antibody for SARS-CoV N protein (prospec) was used for immunohistochemical staining.
Validation	This antibody was been validate in previous publications.<br>PNAS vol 117; pages 16587-16595 (2020) and Nature Medicine vol 28, pages490–495 (2022)

Eukaryotic cell lines

Policy information about cell lines and Sex and Gender in Research

Cell line source(s)	VeroE6/TMPRSS2 cells available at Japanese Collection of Research Bioresources Cell Bank; JCRB 1819. Vero E6-TMPRSS2-T2A-ACE2 cells are available at BEI Resources, NR-54970.
Authentication	Cells are assumed to be authentic by the cell bank or company. No further authentication was performed by the authors.
Mycoplasma contamination	All cell lines are routinely tested and have been negative for mycoplasma.
Commonly misidentified lines (See ICLAC register)	No commonly misidentified cell lines were used in this study.

Animals and other research organisms

Policy information about studies involving animals; ARRIVE guidelines recommended for reporting animal research, and Sex and Gender in Research

Laboratory animals	Six-week-old male wild-type Syrian hamsters (Japan SLC Inc., Shizuoka, Japan) or female 6–8-week-old K18-hACE2 homozygous transgenic hamsters (established colony at UW-Madison from Gilliland T et al. bioRxiv, doi:10.1101/2021.07.26.453840)
Wild animals	N/A
Reporting on sex	Male Syrian hamsters were used along with female and male K18-hACE2 transgenic hamsters were used due to their limited availability. The sex of the hamsters is indicated throughout the manuscript.
Field-collected samples	N/A
Ethics oversight	Animal studies were performed under protocols approved by the Animal Experiment Committee of the Institute of Medical Science, Studies were approved prior by the University of Tokyo (approval number PA19-75) and the Institutional Animal Care and Use Committee at the University of Wisconsin, Madison (protocol number V006426).

Note that full information on the approval of the study protocol must also be provided in the manuscript.
